# Supplementary material for: A human surfactant B deficiency air-liquid interface cell culture model suitable for gene therapy applications
Source: Mol Ther Methods Clin Dev. 2020 Nov 20;20:237–46. doi: 10.1016/j.omtm.2020.11.013 (PMC7782204; doi:10.1016/j.omtm.2020.11.013)
Supplement: Document S1. Figures S1 and S2 [file mmc1.pdf]

**OMTM, Volume *20***

## Supplemental Information

# A human surfactant B deficiency air-liquid interface cell culture model suitable for gene therapy applications

**Altar M. Munis, Stephen C. Hyde, and Deborah R. Gill**

## *Supplementary Materials*

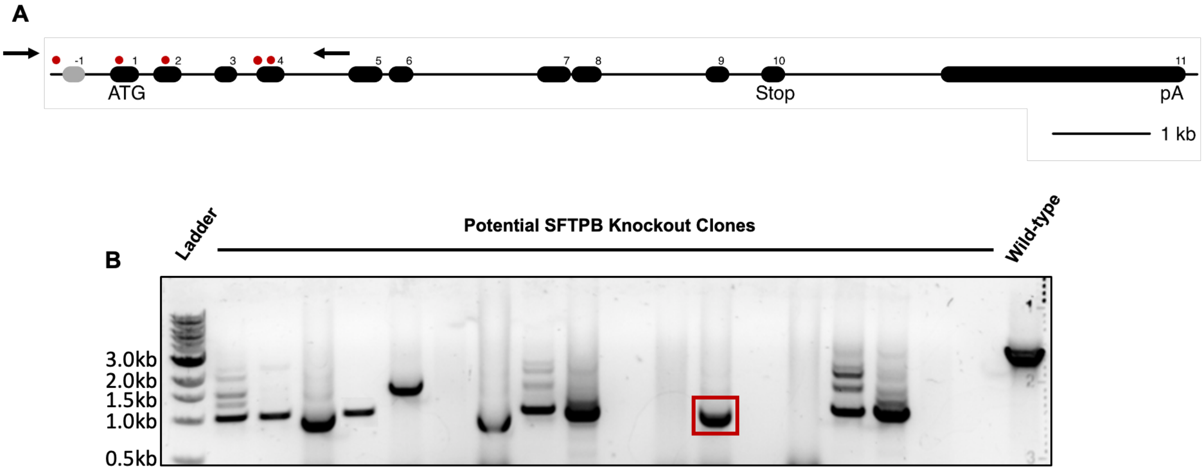


***Supplementary Figure S1: Generation of SFTPB knockout H441 cells.*** **(A)** Schematic of human SFTPB locus. Approximate positions of exons including open reading frame initiation codon (ATG), termination codon (Stop), polyadenylation site (pA) are indicated. In several databases, an N-terminal untranslated sequence is included in the SFTPB locus (e.g. Ensembl transcript IDs ENST00000409383.6 and ENST00000393822.7). This sequence is indicated in gray as exon -1. Guide RNA target locations are indicated with red circles in numerical order (i.e. gRNA1 to gRNA5, left-to-right). The black arrows indicate the approximate locations where the primer pair used in the genotyping polymerase chain reaction bind. **(B)** A representative image of agarose gel electrophoresis following genotyping polymerase chain reaction carried out on potential *SFTPB* gene edited clones. The sizes of the DNA markers of interest are labelled. Sanger sequencing analysis revealed a large deletion encompassing the five chosen gRNAs. By removing exons -1 to 4, the coding sequence of the SPB mature homodimer was completely eliminated. The clone which was selected following sequencing is indicated with a red box. kb: kilobases.

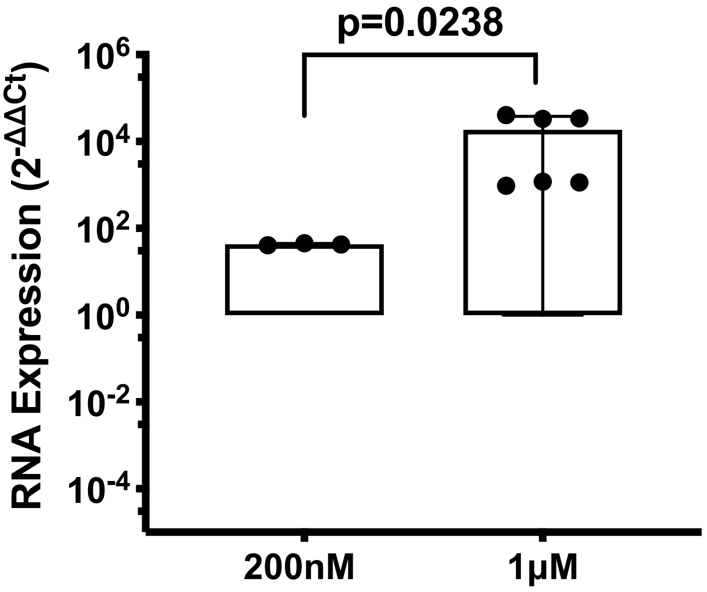


***Supplementary Figure S2: Expression levels of SPB in SALI cultures supplemented with 200nM versus 1μM dexamethasone.*** Each data point represents a biological replicate and columns indicate mean ± SD of data shown. Mann-Whitney test was performed to compare increase in SPB mRNA levels.
