# Supplementary material for: A human surfactant B deficiency air-liquid interface cell culture model suitable for gene therapy applications
Source: Mol Ther Methods Clin Dev. 2020 Nov 20;20:237–46. doi: 10.1016/j.omtm.2020.11.013 (PMC7782204; doi:10.1016/j.omtm.2020.11.013)
Supplement: Document S2. Article plus supplemental information [file mmc2.pdf]

Molecular Therapy
Methods & Clinical Development
Original Article

A human surfactant B deficiency air-liquid interface cell culture model suitable for gene therapy applications

Altar M. Munis,[1] Stephen C. Hyde,[1] and Deborah R. Gill[1]

[1]Gene Medicine Group, Nuffield Division of Clinical Laboratory Sciences, Radcliffe Department of Medicine, University of Oxford, Oxford, UK

Surfactant protein B (SPB) deficiency is a severe monogenic interstitial lung disorder that leads to loss of life in infants as a result of alveolar collapse and respiratory distress syndrome. The development and assessment of curative therapies for the deficiency are limited by the general lack of well-characterized and physiologically relevant *in vitro* models of human lung parenchyma. Here, we describe a new human surfactant air-liquid interface (SALI) culture model based on H441 cells, which successfully recapitulates the key characteristics of human alveolar cells in primary culture as evidenced by RNA and protein expression of alveolar cell markers. SALI cultures were able to develop stratified cellular layers with functional barrier properties that are stable for at least 28 days after air-lift. A *SFTPB* knockout model of SPB deficiency was generated via gene editing of SALI cultures. The *SFTPB*-edited SALI cultures lost expression of SPB completely and showed weaker functional barrier properties. We were able to correct this phenotype via delivery of a lentiviral vector pseudotyped with Sendai virus glycoproteins F/HN expressing SPB. We believe that SALI cultures can serve as an important *in vitro* research tool to study human alveolar epithelium, especially for the development of advanced therapy medicinal products targeting monogenic disorders.

INTRODUCTION

In the lungs, surfactant protein B (SPB) is produced by alveolar type II (ATII) and club cells.[1] This small, hydrophobic protein plays a crucial role in the homeostasis of the alveolar lining fluid, which is responsible for alveolar gas exchange, surfactant maintenance, and immunity against foreign pathogens.[2,3] SPB assists in the packaging and export of pulmonary surfactant from ATII cells directing lung surface tension reduction and allowing alveoli to expand and contract with each breath.[4] In cells, SPB is expressed as a ∼40- to 42-kDa precursor proprotein, which through multiple enzymatic cleavages and disulfide bond formation produces the 16-kDa homodimer mature protein.[1,5,6]

SPB deficiency, first described in 1993, is an autosomal recessive disorder caused by over 30 identified mutations in the *SFTPB* gene.[7,8] The majority of the cases result in loss of life within the first few months of life because patients are in most cases unresponsive to mechanical ventilation, corticosteroid, and exogenous surfactant treatments.[7] The only curative treatment is thought to be lung transplantation; however, the lack of suitable donor organs makes this a non-viable option in most circumstances.

We propose a curative gene therapy approach for the treatment of SPB using our simian immunodeficiency virus (SIV)-based lentiviral vector (LV) pseudotyped with Sendai virus glycoproteins F and HN (rSIV.F/HN).[9] Although non-viral gene therapy approaches have achieved correction of the defect in mouse models,[10,11] the therapeutic effect was short-lived and inefficient. Using rSIV.F/HN, which is optimized for pulmonary gene transfer, curative therapy can be achieved lasting the patient's lifetime or until a suitable donor organ becomes available.

There is, however, a general lack of robust *in vitro* models of the human lung parenchyma to enable high-throughput screening and assessment of either small-molecule or gene therapy approaches. Alveolar epithelium is made up of two major cell types: alveolar type I (ATI) and ATII pneumocytes.[4] Whereas ATI cells are mostly involved in alveolar gas exchange and oxygen uptake, ATII cells, comprising only 5% of the alveolar surface, have progenitor cell characteristics and are responsible for surfactant protein production and secretion.[12] The use of primary human ATII cells *ex vivo* could recapitulate the *in vivo* lung tissue, but such cells are difficult to isolate, not widely available, and can be cultured *in vitro* for only up to two generations because they lose their functional characteristics in culture.[13,14] Recent studies have been able to establish organoid-like spheres from isolated primary human ATII cells, but these require the inclusion of support cells from epithelial or mesenchymal lineages.[15] Furthermore, these ATII alveolosphere cultures do not replicate the structure of the alveolus and show no evidence of cells morphologically resembling or expressing markers of ATI cells. Finally, these methods do not allow for generation of relevant disease models because the cells cannot be reliably expanded in culture following CRISPR-Cas9-based gene manipulations. When

Received 30 June 2020; accepted 17 November 2020;
https://doi.org/10.1016/j.omtm.2020.11.013.

Correspondence: Deborah R. Gill, Gene Medicine Group, Nuffield Division of Clinical Laboratory Sciences, Radcliffe Department of Medicine, University of Oxford, Oxford, UK.
E-mail: deborah.gill@ndcls.ox.ac.uk

This is an open access article under the CC BY license (http://creativecommons.org/licenses/by/4.0/).

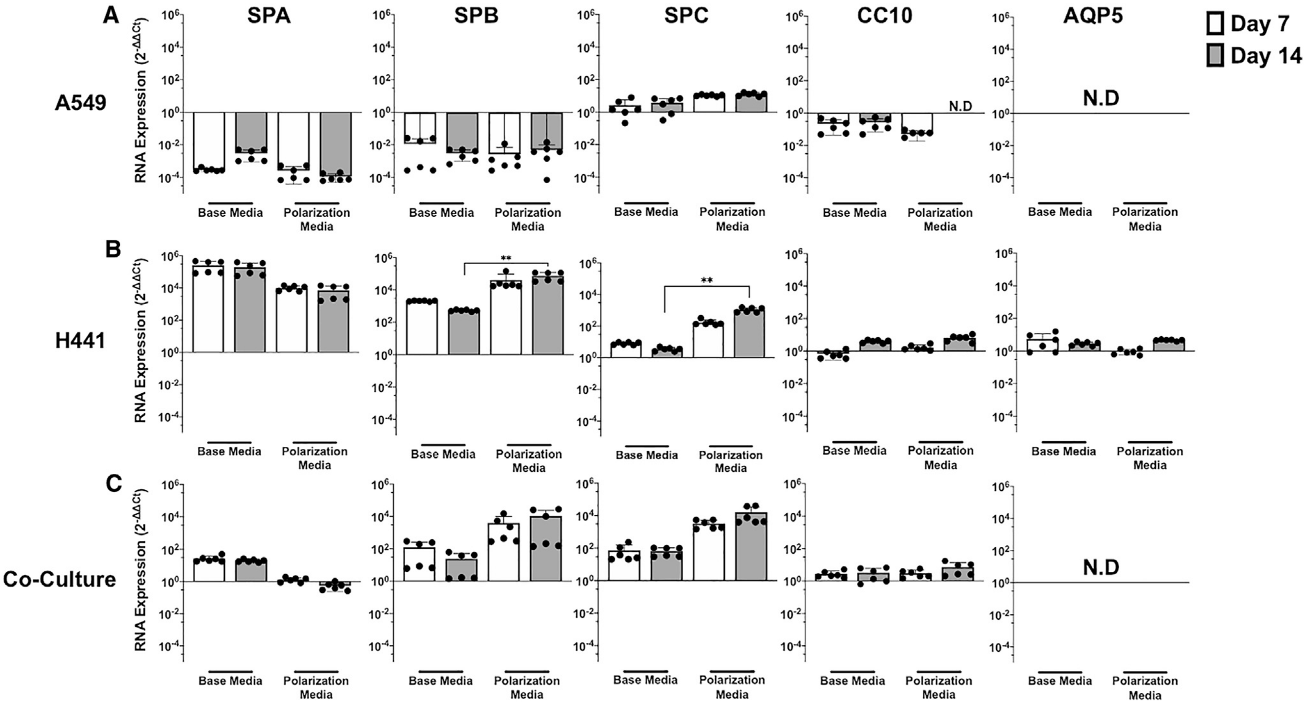

Figure 1. Expression of ATI and ATII cell markers in ALI cultures

(A–C) ALI cultures were established with (A) A549 cells, (B) H441 cells, and (C) co-culture of both cell lines grown in base and polarization media 7 and 14 days after air-lift. Expression levels of SPA, SPB, SPC, CC10, and AQP5 were determined via qRT-PCR and normalized to that of equivalent cells grown in submerged culture using $2^{-\Delta\Delta CT}$ analyses. Each qRT-PCR was performed in three technical replicates, which were averaged for each biological replicate. Each data point represents a biological replicate, and columns indicate mean ± SD of data shown. Mann-Whitney test was performed to compare the increase of SPB and SPC mRNA levels in H441 cells (**$p < 0.01$). ND, not detected.

researchers focused on stem cells as a way forward, they successfully derived lung organoids from human embryonic stem cells and induced pluripotent stem cells that express ATII cell-related surfactant protein markers.[16–18] However, these alveolar or proximal lung organoids[17,19,20] are phenotypically more representative of a developing lung, which makes them unsuitable models for therapeutic assessments unless *in utero* strategies are pursued.

Here, we describe a human surfactant air-liquid interface (SALI) *in vitro* cell culture model based on human pulmonary epithelial H441 cells derived from both ATII and club cell lineages.[21–23] We show that H441 cells, when grown under SALI culture conditions, successfully mimic key characteristics of primary ATII cells. In addition, we carried out analyses on the air-liquid interface (ALI) culture model with regards to functional barrier properties. Finally, using CRISPR-Cas9 gene editing, we generated a SPB deficiency disease model based on SALI cultures and demonstrated correction of the disease phenotype following rSIV.F/HN intervention.

RESULTS

H441 cells demonstrate ATII cell characteristics

A549 and H441 lung adenocarcinoma cells have been widely used as cell culture models for the lung parenchyma in drug discovery and epithelial transport studies,[23–27] and we investigated their potential to serve as a model for surfactant deficiencies. ALI cultures were established from A549 cells, H441 cells, and co-culture of both lines grown in either "base" or "polarization" media.[28] Quantitative reverse-transcription polymerase chain reaction (qRT-PCR) analysis of mRNA expression of cell markers for ATI (aquaporin 5 [AQP5]),[29] ATII (surfactant proteins A [SPA], B [SPB], and C [SPC]),[30] and club (club cell protein 10 [CC10])[31] cells was performed at 1 and 2 weeks following air-lift under the alternate culture media (Figures 1A–1C). qRT-PCR analysis demonstrated a 10,000-fold increase in SPA and SPB expression and a 100-fold increase in SPC expression levels in H441 cells grown as ALI cultures compared with cells grown in submerged culture (Figure 1B). In addition, a modest 5- to 10-fold increase in CC10 and AQP5 mRNA levels was also observed. In contrast, SPA and SPB mRNA levels in A549 cells decreased by 10,000-fold and 1,000-fold, respectively (Figure 1A), and cell proliferation was stunted considerably (data not shown). Despite earlier studies demonstrating statistically significant increases in SPB expression,[32] no synergistic effect of co-culture of the two cell lines was observed on mRNA levels of the cell markers (Figure 1C). Based on the significant differences observed in the expression levels of SPB ($p = 0.0022$) and SPC ($p = 0.0022$) in H441 cells, polarization media were selected for use in further studies.

Furthermore, expression and post-translational processing of SPB and SPC proteins in H441 cells under the new ALI culture conditions

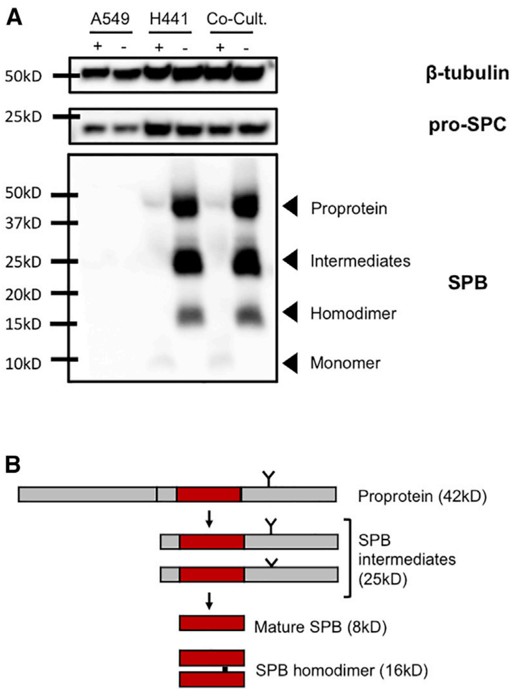

Figure 2. Immunoblot analysis confirms expression of SPB and SPC in SALI cultures

(A) Immunoblot showing SPB and SPC expression in ALI cultures grown in polarization media. Total protein lysates (30 μg) from A549, H441, and co-culture ALI samples were blotted under reducing (+) and non-reducing (–) conditions. β-Tubulin was used as protein loading control and blotted under reducing conditions. The size of the closest protein marker to each target protein is labeled. Data shown are representative of three independent repeats. (B) Schematic representation of the post-translational processing of SPB. N- and C-terminal arms, which undergo protease-mediated cleavage, are shown in gray, while the mature peptide is indicated in red. The single N-linked glycosylation site is marked by a "Y," and the location of the intermolecular disulphide bond unique to mature SPB is indicated with a black bar.

were confirmed via immunoblotting (Figure 2A). Importantly, this protein processing was not observed in H441 cells grown in submerged culture (data not shown). SPB is produced as a 42-kDa proprotein that undergoes protease-mediated processing by napsin and cathepsin H[6] (Figure 2B); these post-translational modifications yield an 8-kDa mature peptide that forms 16-kDa disulfide-linked homodimers prior to being stored in cellular lamellar bodies.[5] The immunoblot analysis (Figure 2A) clearly shows that H441 cells robustly express and correctly process SPB, evident by the presence of the proprotein, intermediates, mature peptide, and the homodimer. Based on these results, the H441 cell line was chosen to establish the SALI cultures.

SALI cultures demonstrate functional barrier properties

To further assess the physiological relevance of SALI culture to the lung parenchyma, we assessed functional barrier properties: transepithelial electrical resistance (TEER) and potential difference (TEPD). While TEER measures the degree of permeability of the ALI culture, TEPD informs about the ability of the cells to maintain an ionic concentration gradient across the culture.[33] Both TEER and TEPD values for the SALI cultures increased following air-lift and stabilized after approximately 7 days (Figures 3A and 3B). TEER measurements reached a maximum of 249.3 ± 25.1 Ωcm^2 and TEPD 1.35 ± 0.13 mV. Both the electrical resistance and potential difference were stable for at least 28 days after air-lift. In contrast, TEER and TEPD values for A549 cells in ALI culture were significantly lower ($p < 0.0001$ and $p = 0.0001$, respectively), remaining around baseline for the duration of the experiment.

The formation of tight junctions in SALI cultures was confirmed via immunocytochemistry against the junction proteins E-cadherin, zona-occludens 1 (ZO-1), claudin-3, claudin-4, claudin-8, and occludin-1 (Figures 3C–3H). While E-cadherin, ZO-1, and claudin-4 were detected on the cell membrane surface, occludin-1, claudin-3, and claudin-8 were localized intracellularly and/or around the nucleus. The expression profiles of these sealing claudins[34,35] were similar to those reported for primary human alveolar cells.[36,37] Based on these results it can be inferred that the SALI procedure results in tightly sealed, polarized cultures with tight junctions present between the cells.

SALI cultures can be efficiently targeted by rSIV.F/HN

Our SIV-based LV platform, pseudotyped with F and HN envelope proteins of Sendai virus, is being developed for the treatment of interstitial lung diseases (ILDs), as well as conducting airway disorders (e.g., cystic fibrosis), and has been shown to efficiently transduce murine lungs and human ALI cell models based on human bronchial epithelial cells.[9] The F/HN pseudotype mediates lentiviral cell entry via sialic acid receptors found abundantly in the lung cells.[38–40] Having established the SALI culture model, we evaluated its suitability for developing gene therapy interventions with our candidate platform. Two weeks after air-lift, SALI cultures were challenged with rSIV.F/HN encoding EGFP, and transduction efficiencies were quantified via fluorescence microscopy and flow cytometry (Figure 4). Cross sections of transduced SALI cultures revealed stratified layers several cells thick (Figure 4A). The rSIV.F/HN was not only able to transduce the surface of the ALI culture efficiently but also achieved penetration of the culture to access cells below. Furthermore, the transduction profile demonstrated strong dose correlation with the number of LV transducing units (TUs) applied to the apical surface of the culture (Figures 4B and 4C). Overall, rSIV.F/HN administration yielded up to 30% EGFP-positive cells at a multiplicity of infection (MOI) of 1.

SPB deficiency model based on SALI cultures can be treated via rSIV.F/HN intervention

We then set out to generate a SPB deficiency disease model based on SALI cultures, using CRISPR-Cas9 gene editing. Five guide RNAs (gRNA) targeting the first four exons of the *SFTPB* locus were inserted into the PX459 V2.0 plasmid.[41] Following five-plasmid co-transfection and puromycin selection, H441 cells were single cell cloned, expanded, and genotyped via PCR (Figure S1), with gene editing confirmed via Sanger sequencing (data not shown). Subsequently,

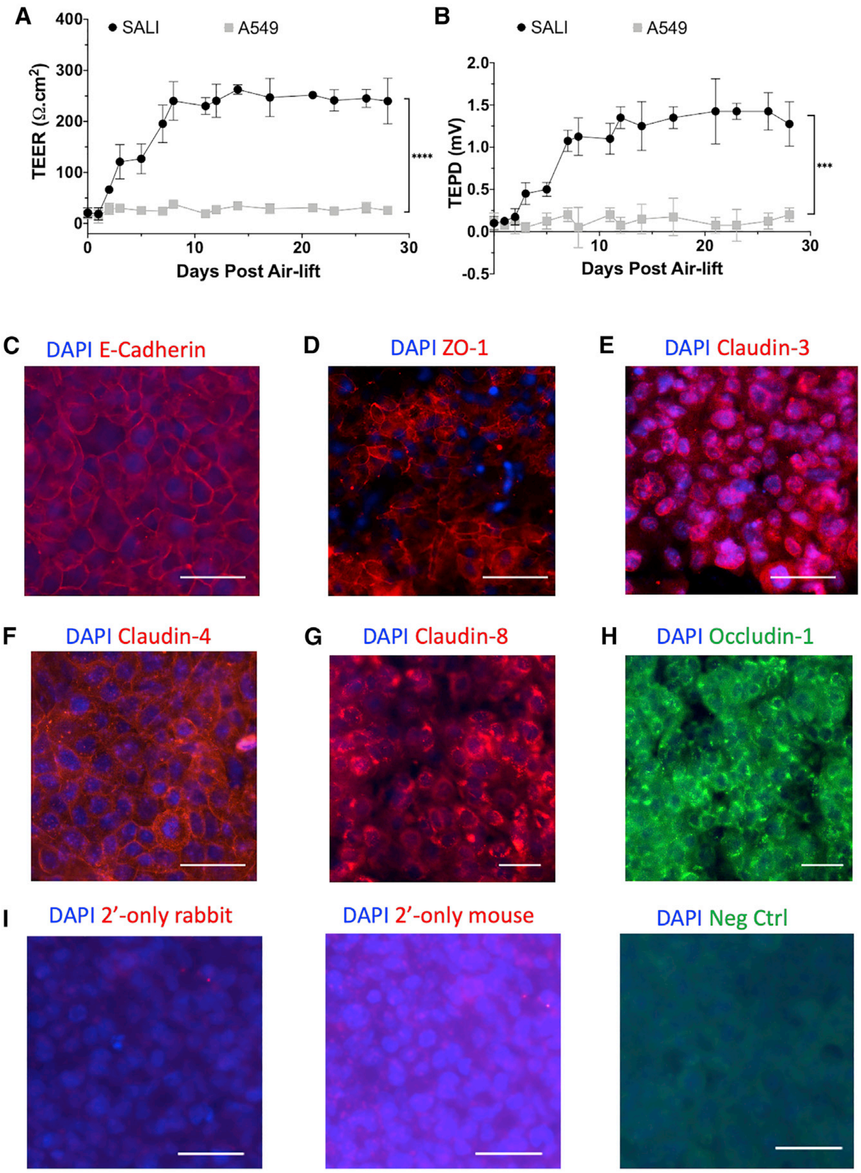

Figure 3. SALI forms cultures with strong barrier properties and tight junction markers

(A and B) TEER (A) and TEPD (B) measurements taken from SALI cultures and A549 cells grown in ALI culture. SALI cultures established strong functional barrier properties approximately 7 days after air-lift with 249.3 ± 25.1 Ωcm^2 TEER and 1.35 ± 0.13 mV TEPD values. Data represent mean ± SD of six biological replicates. Kolmogorov-Smirnov test was performed to compare functional barrier properties of SALI and A549 ALI cultures (***$p = 0.0001$; ****$p < 0.0001$). Immunofluorescence microscopy analyses of tight-junction markers in SALI cultures. (C–H) Expression of (C) E-cadherin, (D) ZO-1, (E) claudin-3, (F) claudin-4, (G) claudin-8, and (H) occludin-1 was detected, confirming the presence of tight junctions in SALI cultures. Representative images from three biological replicates are shown. (I) Staining controls for immunofluorescence microscopy experiments, including secondary-only staining of SALI samples with Alexa Fluor 594-conjugated (left) anti-rabbit and (middle) anti-mouse secondary antibodies used in immunocytochemistry studies, and (right) HEK293T cells were stained with Alexa Fluor 488-conjugated anti-occludin-1 antibody as a negative control. Nuclei are stained blue. Representative images of three biological replicates. All scale bars indicate 50 μm.

SALI cultures were established from the *SFTPB*-edited H441 cells, and SPB expression was assessed via qRT-PCR and immunoblotting to demonstrate loss of SPB expression, thus confirming the *SFTPB* KO status of the SALI culture model (Figures 5B and 6A). In addition, other pulmonary cell markers were investigated to confirm that ATII features of the SALI culture are maintained following gene editing of H441 cells (Figure 5). Although wild-type (WT) and *SFTPB* knockout (KO) SALI cultures demonstrated significantly different RNA expression signatures for SPA, SPB, and SPC ($p < 0.01$), both showed identical expression profiles at the protein level, highlighting that *SFTPB* KO cultures retained their ATII-like characteristics. Interestingly, the KO of the *SFTPB* gene in SALI culture resulted in a significant ($p < 0.0001$) decrease in TEER values, implying an increase in ionic permeability of the ALI culture (Figure 6B).

We aimed to correct the disease phenotype in KO SALI cultures via administration of rSIV.F/HN-expressing SPB protein. Following SALI culture establishment, the cultures were treated with 5×10^6 TUs of rSIV.F/HN encoding either EGFP or SPB (MOI ~1). The treated SALI cultures were grown for a further week, TEER was measured, and cells were subsequently harvested for immunoblotting. Following administration of rSIV.F/HN-expressing SPB, robust expression of mature SPB homodimer was detected in *SFTPB* KO SALI cultures (Figure 6A), and TEER increased to levels comparable with WT SALI cultures ($p = 0.6315$). Crucially, treatment with a control vector expressing EGFP failed to generate SPB homodimers and did not correct the TEER (Figures 6A and 6B, respectively). Together, these results indicate that rSIV.F/HN-expressing SPB can correct the SPB-deficient condition both functionally and phenotypically.

DISCUSSION

In this study, we describe a new *in vitro* cell culture model of the human alveolar epithelium for the study of SPB deficiency and other ILDs. Although primary human alveolar cells would be the gold standard for such models, unlike primary cells or organoids established using alveolar cells,[15] the SALI cultures described here robustly recapitulate expression of ATII cell markers, show reproducible, sustainable long-term culture, are physiologically relevant, and could be used

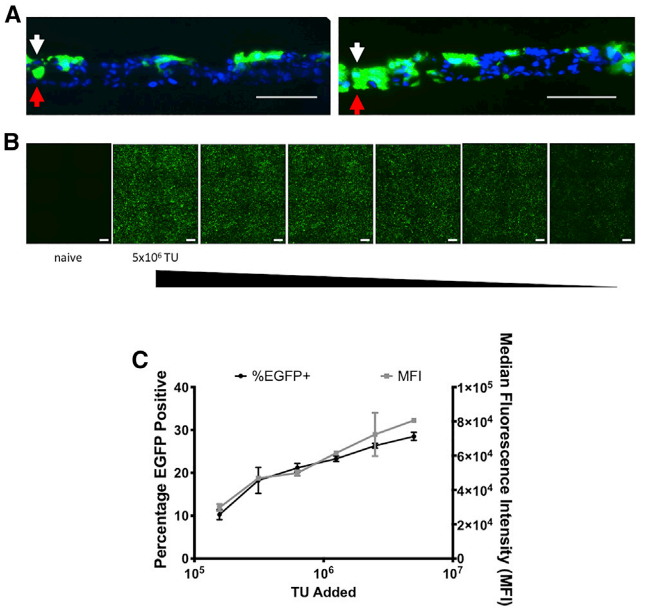

Figure 4. rSIV.F/HN efficiently transduces SALI cultures

SALI cultures were established and 2 weeks after air-lift rSIV.F/HN encoding, enhanced green fluorescent protein encoding (EGFP) was administered apically and used as a measure of transduction efficiency 72 h after transduction. (A) Representative native EGFP fluorescence images of fixed-frozen cryosections of SALI cultures challenged with approximately 1×10^6 transducing units (TUs) of rSIV.F/HN EGFP-positive cells at the apical (white arrows) and basolateral (red arrows) surfaces are shown. Nuclei are stained blue. Multiple layers of stratified cells can be observed in the SALI cross sections, which were effectively targeted by rSIV.F/HN. Scale bar represents 100 μm. The images are representative of six independent images acquired from three biological replicates. (B) Representative *en face* images demonstrating EGFP expression in SALI cultures in response to dilutions of rSIV.F/HN. Scale bars represent 500 μm. The image is representative of images acquired from three biological replicates. (C) Transduction efficiency (i.e., EGFP-positive cell percentage) and median fluorescence intensity (i.e., expression levels) were quantified via flow cytometry. A strong LV dose response was observed in both parameters. Data shown represent mean ± SD of three biological replicates.

for rapid high-throughput screening assessment of therapeutic interventions provided by small molecules, gene therapy, and gene-editing approaches.

Two adenocarcinoma cell lines, H441 and A549 cells, have been widely used to study epithelial drug disposition (reviewed in Sporty et al.[23]) and to assess epithelial sodium[42] and chloride[43] channel functions and water transport characteristics of the alveolar epithelium.[44] Therefore, we investigated these two cell lines for their potential to mimic the lung parenchyma while focusing on the disease characteristics of SPB deficiency. The ability of H441 cells to grow in Transwells under ALI or liquid-covered culture conditions has been previously established,[27] and the use of polarization media for H441 cells to achieve a tighter and more polarized cell monolayer has been described.[28] In line with published findings, we observed that, unlike A549 cells, H441 cells grown in ALI culture conditions can proliferate robustly and replicate phenotypic and functional properties of primary ATII cells, as evidenced by the expression of unique surfactant proteins (Figure 1). Our data demonstrated that a variation of the polarization media (1 μM versus 200 nM dexamethasone; Figure S2) for H441 cells resulted in significantly higher levels of SPB and SPC mRNA expression compared with cells grown in base media (Figure 1). In addition, we also observed that H441 cells in SALI culture formed stratified cell layers able to develop and maintain TEER and TEPD, similar to that of primary human ATII cells in culture,[13] indicating formation of tight junctions between cells (Figure 3). This was confirmed by immunocytochemistry analysis of cultures that revealed expression of seal-forming claudin-3 and -4, as well as other tight junction proteins E-cadherin, ZO-1, and occludin-1. In contrast, but similar to previous studies, A549 cells did not express characteristic surfactant proteins and could not form polarized ALI cultures, highlighting an inability, under the chosen culture conditions, to form functional tight junctions.[45–47]

Having established the principle of SALI cultures to model the lung parenchyma, we set out to generate a SPB deficiency disease model. To treat SPB deficiency, we aim to use a targeted gene therapy approach using our rSIV.F/HN lentiviral platform, which is highly efficient in transducing murine conducting airways, as well as lung parenchyma.[9] A crucial use of the SALI model would be high-throughput screening of alternative vector configurations (e.g., promoters, transgenes, etc.) for the selection of a lead candidate; therefore, we confirmed that our pseudotyped rSIV.F/HN vector was able to efficiently target and transduce SALI cultures (Figure 4). Approximately 30% of EGFP-positive cells were observed, along with a modest level of cell layer penetration by rSIV.F/HN. The latter observation could be perceived as an indication of inadequate tight junction formation; however, staining of tight junction proteins in cross sections of transduced SALI cultures revealed that under ALI conditions, H441 cells do not grow in uniform stratified monolayers as evidenced by DAPI-negative pockets in individual planes of focus (Figure 3) and SALI cross sections (Figure 4A). In contrast, ALI cultures formed using primary human cells establish pseudostratified cell layers, which are terminally differentiated and quiescent on the apical side but show growth on the basolateral side.[48]

We exploited CRISPR-Cas9 technology to generate *SFTPB* KO SALI cultures, with *SFTPB*-edited cells showing complete loss of SPB expression in immunoblots (Figure 6). Interestingly, *SFTPB* KO also affected the permeability of the ALI cultures formed. We speculate that the increased permeability is caused by a disruption in SPB regulation of the surface liquid resulting in changes to the surface tension on the apical side of the ALI cultures. It is known that SPB plays an important role in establishment and maintenance of lipid bilayers in the lung through the generation of tubular myelin,[49,50] and it is possible that the lack of SPB expression also may have led to disruption of this surfactant-based macrostructure and lipid bilayers, resulting in poorly formed SALI cultures. No distinct phenotypic differences in the lamellar body-like acidic compartments were observed between WT and *SFTPB* KO SALI cultures as measured by the ATII cell lamellar body dye LysoTracker[51] and fluorescent membrane

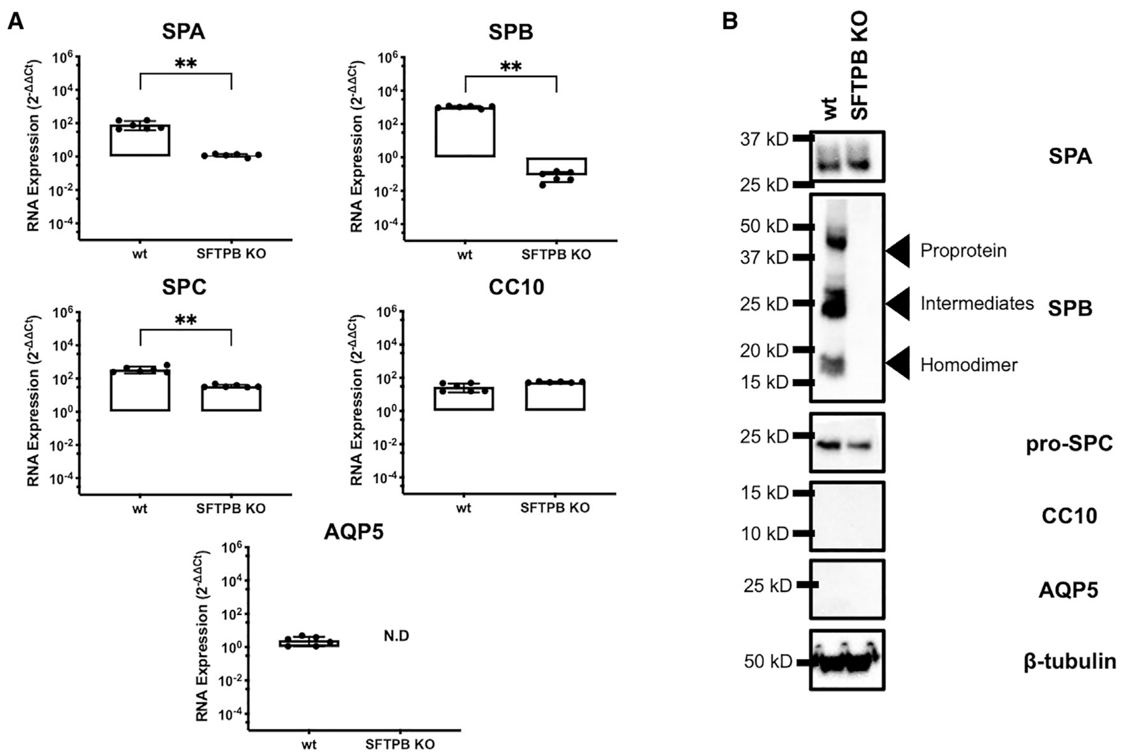

Figure 5. Comparison of RNA and protein expression signatures of WT and *SFTPB* KO SALI cultures

WT and *SFTPB* KO SALI cultures were grown in parallel in polarization medium. (A and B) 14 days after air-lift expression of SPA, SPB, SPC, CC10, and AQP5 was assessed at (A) RNA and (B) protein levels via qRT-PCR and immunoblotting, respectively. Each qRT-PCR was performed in three technical replicates, which were averaged for each biological replicate. Each data point represents a biological replicate, and columns indicate mean ± SD of data shown. Mann-Whitney test was performed to compare differences in mRNA levels (** $p < 0.01$). For immunoblotting experiments, data shown are representative of three independent repeats. The size of the closest protein marker to each target protein is labeled.

probe LAURDAN[52] (data not shown). The application of rSIV.F/HN to the apical surface of the SALI cultures fully restored both SPB expression and the TEER levels of the *SFTPB* KO SALI cultures (Figure 6); together, these observations highlight the suitability of SALI cultures as a model of ILD for gene therapy and potentially other applications. Altogether, the observation that SALI cultures express other ATII cell markers (e.g., SPA, SPC) and the ability of H441 cells to model ion transport[28] suggest the SALI model could be useful for the study of other diseases of surfactant metabolism (related to *SFTPC* or ATP-binding cassette sub-family A member 3 [*ABCA3*] genes) and other ILDs, such as pulmonary alveolar microlithiasis caused by inadequate phosphate clearance[53] (related to solute carrier family 34 member 2 [*SLC34A2*] gene).

The main limitation of the SALI model is that it is based on H441 cells: an adenocarcinoma cell line with a hyper-diploid karyotype and modal number of 52 chromosomes[21] (http://www.atcc.org/Products/All/HTB-174.aspx?geo_country=us). Based on COSMIC Cell Line Gene Mutation Profiles, there are 663 gene mutations identified in H441 cells,[54,55] which do not include any of the surfactant protein loci. Depending on the gene of interest, these characteristics of H441 cells may make SALI cultures less suitable for applications involving *in vitro* gene-editing screening and approaches. To mitigate this, prior genotyping analysis may need to be conducted to determine the number of copies and integrity of the target locus and/or gene of interest prior to applications using Cas9-mediated homology-directed repair and homology-independent targeted gene integration.

In conclusion, here we describe a new *in vitro* cell culture model that robustly recapitulates human ATII cells in primary cell culture. In addition to offering several practical advantages over existing primary cell and stem-cell-based models, SALI cultures can be manipulated by Cas9-based gene editing to create disease models of the lung parenchyma. We anticipate that use of the human SALI culture model will supplement murine and other animal *in vivo* studies, constituting a valuable research tool in the development of advanced therapy medicinal products targeting human lung alveolar epithelium.

MATERIALS AND METHODS

Cells used

All cell lines were obtained from the American Type Culture Collection (ATCC). NCI-H441 (#HTB-174, referred to as H441; ATCC) cells were maintained in RPMI-1640 (#A1049101; GIBCO) medium

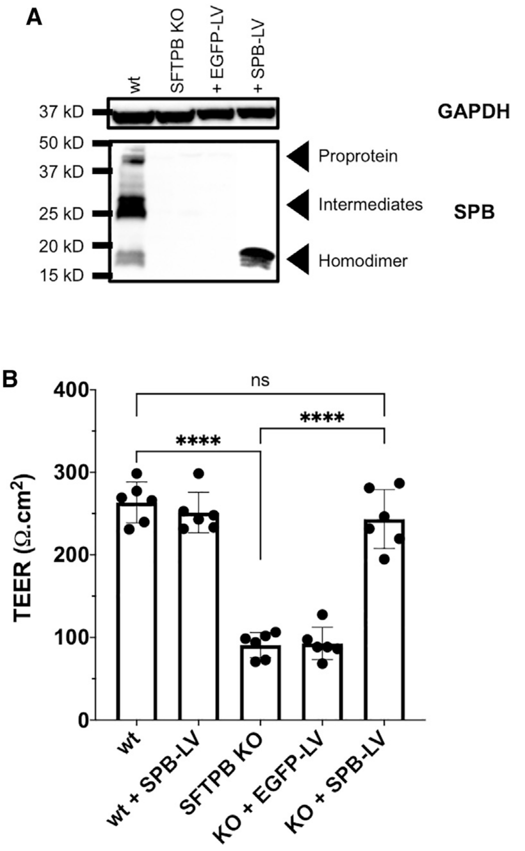

Figure 6. Functional and phenotypical correction of *SFTPB* KO SALI cultures following administration of rSIV.F/HN-expressing SPB

WT and *SFTPB* KO SALI cultures were transduced with approximately 1 $\times$ 10^6 TUs rSIV.F/HN encoding SPB or EGFP. (A) Immunoblot showing expression of mature SPB homodimer following rSIV.F/HN administration. Total protein lysates (30 μg) from WT, treated, and untreated *SFTPB* KO SALI samples were blotted under non-reducing conditions to detect SPB expression. GAPDH was used as protein loading control and blotted under reducing conditions. The size of the closest protein marker to each target protein is labeled. Data shown are representative of three independent repeats performed. (B) Correction of functional barrier properties lost due to *SFTPB* KO in SALI samples. As a result of *SFTPB* KO, a statistically significant decrease in TEER was observed in SALI cultures from 263.6 ± 24.8 Ωcm^2 to 90.7 ± 15.3 Ωcm^2 ($p < 0.0001$). This was corrected back to WT levels, 243.4 ± 35.7 Ωcm^2, following delivery of rSIV.F/HN-expressing SPB. Each data point represents a biological replicate, and columns indicate mean ± SD of date shown. One-way ANOVA with Tukey's multiple comparison test was performed for statistical analyses (****$p < 0.0001$). ns, not significant.

supplemented with 50 U/mL penicillin-50 mg/mL streptomycin (GIBCO) and 10% fetal calf serum (FCS; Sigma). A549 (#CCL-185; ATCC) cells were maintained in Ham's F-12K (#21127022; GIBCO) medium supplemented with 2 mM L-glutamine (GIBCO), 50 U/mL penicillin-50 mg/mL streptomycin (GIBCO), and 10% FCS (Sigma). These maintenance media are referred to as "base" media throughout the manuscript. All cell lines were cultured in a humidified environment at 37°C and 5% CO_2. Cell passages 6–18 for NCI-H441 cells and 10–23 for A549 cells were used for this study.

Establishment of ALI cultures

ALI cultures were established by culturing cells in 12-well Transwell inserts (Corning). Approximately 1 $\times$ 10^5 cells/well in base media (5 $\times$ 10^4 each of H441 and A549 cells in the case of co-culture) were seeded into Transwells and allowed to attach and proliferate for 48 h. On day 3, the medium on the apical side of the Transwell chamber was removed to air-lift the cells, and the medium on the basolateral side of the chamber was replaced with either "base" medium or "polarization" medium.[28] The polarization medium comprised either RPMI-1640 (H441s cells and co-culture) or F12-K (A549s) supplemented with 2 mM L-glutamine, 50 U/mL penicillin, 50 mg/mL streptomycin, 1% insulin-transferrin-selenium (GIBCO), 4% FCS, and 1 μM dexamethasone (Sigma). Media were subsequently changed three times per week throughout the described experiments. Cells were grown under SALI conditions for 14 days after air-lift prior to experimentation unless otherwise stated.

qRT-PCR

qRT-PCR was used to determine RNA expression of several ATI and ATII cell markers in established ALI cultures. RNA was extracted from ALI cultures using a QIAGEN RNeasy Mini Kit (QIAGEN). On-column DNase I digest was performed to prevent genomic DNA carry-over according to manufacturer's instructions. qRT-PCR was carried out directly on extracted RNA samples using Power SYBR Green RNA-to-CT kit (Applied Biosystems) in a QuantStudio 7 Flex Real Time PCR System (Thermo Scientific). The thermal dissociation protocol was run at the end of the PCR to assess the specificity of products that were present in the reaction. Target genes with $C_T \geq 40$ were considered undetectable. The $2^{-\Delta\Delta CT}$ method was utilized to determine relative expression levels of target genes. β-Actin was used as the reference gene, and expression was normalized to that of cells grown in submerged culture for each relevant ALI sample. All qRT-PCR assays were carried out in three technical repeats. Primers used were as follows: SPA forward [FW]: 5′-TTGGAGCCT GAAAAGAAGGA-3′, reverse [RS]: 5′-GGCTTGGAGCTCCTCAT CTA-3′; SPB FW: 5′-AATTCCCCATTCCTCTCCCCTAT-3′, RS: 5′-GATGCCGCCCGCCAC-3′; SPC FW: 5′-AGCCAGAAACACAC GGAGATGGTT-3′, RS: 5′-ATCTTCATGATGTAGCAGCAGGT GCC-3′; CC10 FW: 5′-AGCATCATTAAGCTCATGGAAAAA-3′, RS: 5′-GTGGACTCAAAGCATGGCAG-3′; AQP5 FW: 5′-CAACAA CAACACAACG-3′, RS: 5′-TAGATTCCGACAAGGT-3′; β-actin FW: 5′-AGAGCTACGAGCTGCCTGAC-3′, RS: 5′-AGCACTGTGT TGGCGTACAG-3′.

Immunoblotting

Immunoblotting was used to confirm functional *SFTPB* KO of H441 cell clones, as well as to investigate expression of several ATI and ATII cell markers in SALI cultures. In brief, cells were suspended in a lysis buffer (25 mM Tris-HCl [pH 7.5], 150 mM NaCl, 1% v/v Triton X-100) supplemented with cOmplete mini protease inhibitor cocktail (Roche). Total protein concentrations were determined using the Pierce BCA Protein Assay Kit (Thermo Scientific) according to the manufacturer's instructions. Prior to sodium dodecyl sulfate polyacrylamide gel electrophoresis (SDS-PAGE), samples were incubated

at 95°C for 5 min in Laemmli sample buffer (Bio-Rad) for non-reducing conditions or Laemmli buffer supplemented with 2-mercaptoethanol for reducing conditions. Total protein samples (30 μg) were separated on 12% NuPAGE Bis-Tris precast protein gels (Invitrogen) and transferred onto 0.2-μm nitrocellulose membranes (Bio-Rad). Membranes were blocked in 5% blotting-grade blocker (Bio-Rad) in phosphate-buffered saline (PBS; Sigma) supplemented with 0.1% Tween 20 (Sigma) for 1 h at room temperature. Membranes were then incubated with primary antibodies overnight at 4°C followed by the appropriate horseradish peroxidase (HRP)-conjugated secondary antibody for 1 h at room temperature. Bands were visualized using the Clarity Western ECL Substrate kit (Bio-Rad), and images were captured using the iBright FL1500 imaging system (Invitrogen). Primary and secondary antibodies used were as follows: rabbit anti-SPB (1:5,000, #WRAB-48604; Seven Hills Bioreagents), rabbit anti-pro-SPC (1:500, #AB3786; Sigma), mouse anti-SPA (1:1,000, #ab51891; Abcam), rabbit anti-AQP5 (1:1,000, #ab92320; Abcam), rabbit-anti-uteroglobin (1:500, #ab40873; Abcam), rabbit anti-β-tubulin (1:500, #ab6046; Abcam), rabbit anti-GAPDH (1:1,000, #G9545; Sigma), swine anti-rabbit IgG (1:5,000, #P0399; Dako), and goat anti-mouse IgG (1:2,000, #ab205719; Abcam).

Lentiviral production and transduction

Production of recombinant SIV vectors was performed using the five-plasmid transient transfection method.[9] In brief, the LV-MAX Lentiviral Productions System was utilized per manufacturer's instructions. Media containing LVs were harvested 48 h post-transfection and purified via anion exchange chromatography and concentrated by tangential flow filtration. The vectors were formulated in TSSM (Tris, sodium chloride, sucrose, and mannitol buffer),[56] aliquoted, and stored at −80°C.

Functional titers were determined via real-time PCR following transduction of LV-MAX cells with serial dilutions of the LV. Primers against WPRE (woodchuck hepatitis virus post-transcriptional regulatory element; vector target) (FW: 5′-TGGCGTGGTGTGCACT GT-3′; RS: 5′-CCCGGAAAGGAGCTGACA-3′; Probe: 5′-FAM-TT GCTGACGCAACCCCCACTGG-TAMRA-3′) and hCFTR (human cystic fibrosis transmembrane conductance regulator; endogenous control) (FW: 5′-CTTCCCCCATCTTGGTTGTTC-3′; RS: 5′-TGAC AGTTGACAATGAAGATAAAGATGA-3′; Probe: 5′-VIC-TGTC CCCATTCCAGCCATTTGTATCCT-TAMRA-3′) were used.

SALI cultures were challenged with LV via administration to the apical side of the culture and after 6 h, the vector was removed. Approximately 72 h post-transduction, relevant protocols were carried out for fluorescence imaging, TEER and TEPD measurements, and immunoblotting.

Immunofluorescence microscopy

Cells in Transwell inserts were fixed with 4% paraformaldehyde (PFA) in PBS for 15 min at room temperature. Cells were then washed twice with PBS and dehydrated in a solution of 30% sucrose in PBS overnight at 4°C. The membranes from the inserts, with fixed and dehydrated cells attached, were removed with a scalpel and transferred to 24-well plates for free-floating staining. Cells were then permeabilized with 1% bovine serum albumin (BSA) in PBS supplemented with 0.1% Triton X-100 for 15 min at room temperature, blocked with 1% BSA in PBS for 1 h at room temperature, and then incubated with primary antibodies overnight at 4°C. Following three washes with PBS, cells were incubated with appropriate secondary antibodies for 1 h at room temperature and mounted on glass slides using ProLong Gold Antifade Mountant with DAPI. Cells were imaged using the EVOS FL Auto 2 Imaging System (Invitrogen). Laser settings were optimized to acquire best signal for each target, and no specific staining was observed in secondary-only staining controls. Images were processed and analyzed using ImageJ software (NIH). Antibodies used were as follows: E-cadherin (1:500, mouse, #13-1700; Invitrogen), ZO-1 (1:100, rabbit, #40-2200; Invitrogen), claudin-3 (1:100, rabbit, #34-1700; Invitrogen), claudin-4 (1:100, mouse, #32-9400; Invitrogen), claudin-8 (1:100, rabbit, #710222; Invitrogen), occludin-1 (1:100, Alexa Fluor 488 conjugated, mouse, #331588; Invitrogen), donkey anti-rabbit IgG, Alexa Fluor 594 conjugated (1:500, #A-21207; Invitrogen), and goat anti-mouse IgG, Alexa Fluor 594 conjugated (1:500, #A-11005; Invitrogen).

To analyze SALI samples transduced with vector encoding EGFP, 72 h post-transduction cells were fixed with 4% PFA in PBS, dehydrated using a 30% sucrose in PBS solution, embedded in 1:1 v/v solution of optimal cutting temperature (OCT) compound and 30% sucrose/PBS, and cryopreserved at −80°C. For immunofluorescence analysis, embedded SALI samples were cryosectioned using a Cryo Star NX70 (Thermo Scientific) cryostat at 7 μm thickness and mounted on glass slides using ProLong Gold Antifade Mountant with DAPI (Invitrogen). Native fluorescence was acquired using EVOS FL Auto 2 Imaging System (Invitrogen). Images were processed and analyzed using ImageJ software (NIH).

TEER and TEPD measurements

TEER and TEPD measurements were performed to assess the barrier function of SALI cultures. Millicell ERS-2 Voltohmmeter (Millipore) was used to measure TEER and TEPD values following the manufacturer's instructions. In brief, pre-warmed (to room temperature) PBS solution was added to both the apical (0.5 mL) and basolateral (1 mL) side of the Transwell culture chamber. Cells were equilibrated with PBS for 15 min at room temperature prior to the taking of measurement using the electrodes. Measurements taken from blank inserts without cells were regarded as background. TEPD and TEER values were calculated via the following formulas:

$$TEPD(mV) = mV_{sample} - mV_{blank}$$

$$TEER(\Omega.cm^2) = \left(\Omega_{sample} - \Omega_{blank}\right) x\ Surface\ Area_{insert}$$

Flow cytometry

Flow cytometry analysis was used to determine LV transduction efficiency of SALI cultures. In brief, 72 h post-transduction, cells were

harvested, washed twice with PBS, and fixed in 2% PFA/PBS for 15 min at room temperature. Following two further PBS washes, cells were analyzed to determine percentages of EGFP-positive cells and median fluorescence intensity values. All experiments were carried out using BD LSR II (BD Biosciences) machine and FACS Diva analysis software (BD Biosciences). Data analysis was carried out using FlowJo single-cell analysis software (BD Biosciences).

Generation of *SFTPB* knockouts

CRISPR-Cas9 technology was utilized to generate the *SFTPB* knockout H441 cell line. Five gRNAs were designed targeting the first four exons of the *SFTPB* locus (gRNA1: 5′-GTAGTGCTTCCCGAG TATAG-3′, gRNA2: 5′-GGTGCATGGCCCCTTATAGC-3′, gRNA3: 5′-TTCCTGTAGGCAATGCCCT-3′, gRNA4: 5′-GCTTGACGACT ACTTCCCCC-3′, gRNA5: 5′-CCTGGTCATCGACTACTTC-3′) (Figure S1) and sub-cloned into pSpCas9(BB)-2A-Puro (PX459) V2.0 (#62988; Addgene).[41] Five-plasmid co-transfection was carried out using Lipofectamine 3000 (Invitrogen) according to the manufacturer's instructions. 24 h post-transfection, cells were placed under antibiotic selection with 2.5 μg/mL puromycin (Sigma). 48 h later, cells were harvested and seeded into six-well culture plates in 50% v/v H441 maintenance media and Cultrex Reduced Growth Factor Basement Membrane Extract (BME) (Trevigen) mixture at a 1×10^3 cells/mL density. Cells were cultured in 50% conditioned media until single-cell colonies formed (approximately 1 week). Three weeks after seeding, clones were recovered from BME using Cell Recovery Solution (Corning) and scaled up to 12-well plates. Gene editing in each clone was confirmed via PCR and Sanger sequencing (Figure S1).

Statistical analyses

All statistical analyses were performed using GraphPad Prism 8 software. Details of all tests, including the calculated p values, are indicated in figure legends.

SUPPLEMENTAL INFORMATION

Supplemental Information can be found online at https://doi.org/10.1016/j.omtm.2020.11.013.

ACKNOWLEDGMENTS

A.M.M. is supported by Wellcome Trust Portfolio grant 110579/Z/15/Z (to D.R.G. and S.C.H.).

AUTHOR CONTRIBUTIONS

A.M.M. designed and performed experiments, analyzed the presented data, and prepared the manuscript. S.C.H. and D.R.G. supervised the study. All authors reviewed the manuscript.

DECLARATION OF INTERESTS

D.R.G. and S.C.H. hold patents on F/HN LV technology. The authors have no other relevant affiliations or financial involvement with any organization or entity with a financial interest in or financial conflict with the subject matter or materials discussed in the manuscript, apart from those disclosed.

REFERENCES

1. Weaver, T.E. (1998). Synthesis, processing and secretion of surfactant proteins B and C. Biochim. Biophys. Acta *1408*, 173–179.
2. Hollenhorst, M.I., Richter, K., and Fronius, M. (2011). Ion transport by pulmonary epithelia. J. Biomed. Biotechnol. *2011*, 174306.
3. Wert, S.E., Whitsett, J.A., and Nogee, L.M. (2009). Genetic disorders of surfactant dysfunction. Pediatr. Dev. Pathol. *12*, 253–274.
4. Knudsen, L., and Ochs, M. (2018). The micromechanics of lung alveoli: structure and function of surfactant and tissue components. Histochem. Cell Biol. *150*, 661–676.
5. Korimilli, A., Gonzales, L.W., and Guttentag, S.H. (2000). Intracellular localization of processing events in human surfactant protein B biosynthesis. J. Biol. Chem. *275*, 8672–8679.
6. Ueno, T., Linder, S., Na, C.L., Rice, W.R., Johansson, J., and Weaver, T.E. (2004). Processing of pulmonary surfactant protein B by napsin and cathepsin H. J. Biol. Chem. *279*, 16178–16184.
7. Nogee, L.M., de Mello, D.E., Dehner, L.P., and Colten, H.R. (1993). Brief report: deficiency of pulmonary surfactant protein B in congenital alveolar proteinosis. N. Engl. J. Med. *328*, 406–410.
8. Tredano, M., van Elburg, R.M., Kaspers, A.G., Zimmermann, L.J., Houdayer, C., Aymard, P., Hull, W.M., Whitsett, J.A., Elion, J., Griese, M., and Bahuau, M. (1999). Compound SFTPB 1549C–>GAA (121ins2) and 457delC heterozygosity in severe congenital lung disease and surfactant protein B (SP-B) deficiency. Hum. Mutat. *14*, 502–509.
9. Alton, E.W.F.W., Beekman, J.M., Boyd, A.C., Brand, J., Carlon, M.S., Connolly, M.M., Chan, M., Conlon, S., Davidson, H.E., Davies, J.C., et al. (2017). Preparation for a first-in-man lentivirus trial in patients with cystic fibrosis. Thorax *72*, 137–147.
10. Barnett, R.C., Lin, X., Barravecchia, M., Norman, R.A., de Mesy Bentley, K.L., Fazal, F., Young, J.L., and Dean, D.A. (2017). Featured Article: Electroporation-mediated gene delivery of surfactant protein B (SP-B) restores expression and improves survival in mouse model of SP-B deficiency. Exp. Biol. Med. (Maywood) *242*, 1345–1354.
11. Kormann, M.S.D., Hasenpusch, G., Aneja, M.K., Nica, G., Flemmer, A.W., Herber-Jonat, S., Huppmann, M., Mays, L.E., Illenyi, M., Schams, A., et al. (2011). Expression of therapeutic proteins after delivery of chemically modified mRNA in mice. Nat. Biotechnol. *29*, 154–157.
12. Iwasaki, A., Foxman, E.F., and Molony, R.D. (2017). Early local immune defences in the respiratory tract. Nat. Rev. Immunol. *17*, 7–20.
13. Bove, P.F., Dang, H., Cheluvaraju, C., Jones, L.C., Liu, X., O'Neal, W.K., Randell, S.H., Schlegel, R., and Boucher, R.C. (2014). Breaking the in vitro alveolar type II cell proliferation barrier while retaining ion transport properties. Am. J. Respir. Cell Mol. Biol. *50*, 767–776.
14. Dobbs, L.G., and Johnson, M.D. (2007). Alveolar epithelial transport in the adult lung. Respir. Physiol. Neurobiol. *159*, 283–300.
15. Barkauskas, C.E., Chung, M.I., Fioret, B., Gao, X., Katsura, H., and Hogan, B.L. (2017). Lung organoids: current uses and future promise. Development *144*, 986–997.
16. Cerrada, A., de la Torre, P., Grande, J., Haller, T., Flores, A.I., and Pérez-Gil, J. (2014). Human decidua-derived mesenchymal stem cells differentiate into functional alveolar type II-like cells that synthesize and secrete pulmonary surfactant complexes. PLoS ONE *9*, e110195.
17. Huang, S.X., Islam, M.N., O'Neill, J., Hu, Z., Yang, Y.G., Chen, Y.W., Mumau, M., Green, M.D., Vunjak-Novakovic, G., Bhattacharya, J., and Snoeck, H.W. (2014). Efficient generation of lung and airway epithelial cells from human pluripotent stem cells. Nat. Biotechnol. *32*, 84–91.
18. Leibel, S., and Post, M. (2016). Endogenous and Exogenous Stem/Progenitor Cells in the Lung and Their Role in the Pathogenesis and Treatment of Pediatric Lung Disease. Front Pediatr. *4*, 36.
19. Chen, Y.W., Huang, S.X., de Carvalho, A.L.R.T., Ho, S.H., Islam, M.N., Volpi, S., Notarangelo, L.D., Ciancanelli, M., Casanova, J.L., Bhattacharya, J., et al. (2017). A three-dimensional model of human lung development and disease from pluripotent stem cells. Nat. Cell Biol. *19*, 542–549.
20. Jacob, A., Vedaie, M., Roberts, D.A., Thomas, D.C., Villacorta-Martin, C., Alysandratos, K.D., Hawkins, F., and Kotton, D.N. (2019). Derivation of

self-renewing lung alveolar epithelial type II cells from human pluripotent stem cells. Nat. Protoc. *14*, 3303–3332.

21. Gazdar, A.F., Linnoila, R.I., Kurita, Y., Oie, H.K., Mulshine, J.L., Clark, J.C., and Whitsett, J.A. (1990). Peripheral airway cell differentiation in human lung cancer cell lines. Cancer Res. *50*, 5481–5487.

22. Rehan, V.K., Torday, J.S., Peleg, S., Gennaro, L., Vouros, P., Padbury, J., Rao, D.S., and Reddy, G.S. (2002). 1Alpha,25-dihydroxy-3-epi-vitamin D3, a natural metabolite of 1alpha,25-dihydroxy vitamin D3: production and biological activity studies in pulmonary alveolar type II cells. Mol. Genet. Metab. *76*, 46–56.

23. Sporty, J.L., Horálková, L., and Ehrhardt, C. (2008). In vitro cell culture models for the assessment of pulmonary drug disposition. Expert Opin. Drug Metab. Toxicol. *4*, 333–345.

24. Foster, K.A., Oster, C.G., Mayer, M.M., Avery, M.L., and Audus, K.L. (1998). Characterization of the A549 cell line as a type II pulmonary epithelial cell model for drug metabolism. Exp. Cell Res. *243*, 359–366.

25. Hermanns, M.I., Unger, R.E., Kehe, K., Peters, K., and Kirkpatrick, C.J. (2004). Lung epithelial cell lines in coculture with human pulmonary microvascular endothelial cells: development of an alveolo-capillary barrier in vitro. Lab. Invest. *84*, 736–752.

26. Rucka, Z., Vanhara, P., Koutna, I., Tesarova, L., Potesilova, M., Stejskal, S., Simara, P., Dolezel, J., Zvonicek, V., Coufal, O., and Capov, I. (2013). Differential effects of insulin and dexamethasone on pulmonary surfactant-associated genes and proteins in A549 and H441 cells and lung tissue. Int. J. Mol. Med. *32*, 211–218.

27. Salomon, J.J., Muchitsch, V.E., Gausterer, J.C., Schwagerus, E., Huwer, H., Daum, N., Lehr, C.M., and Ehrhardt, C. (2014). The cell line NCl-H441 is a useful in vitro model for transport studies of human distal lung epithelial barrier. Mol. Pharm. *11*, 995–1006.

28. Ren, H., Birch, N.P., and Suresh, V. (2016). An Optimised Human Cell Culture Model for Alveolar Epithelial Transport. PLoS ONE *11*, e0165225.

29. Wang, Y., Tang, Z., Huang, H., Li, J., Wang, Z., Yu, Y., Zhang, C., Li, J., Dai, H., Wang, F., et al. (2018). Pulmonary alveolar type I cell population consists of two distinct subtypes that differ in cell fate. Proc. Natl. Acad. Sci. USA *115*, 2407–2412.

30. Fehrenbach, H. (2001). Alveolar epithelial type II cell: defender of the alveolus revisited. Respir. Res. *2*, 33–46.

31. Laucho-Contreras, M.E., Polverino, F., Tesfaigzi, Y., Pilon, A., Celli, B.R., and Owen, C.A. (2016). Club Cell Protein 16 (CC16) Augmentation: A Potential Disease-modifying Approach for Chronic Obstructive Pulmonary Disease (COPD). Expert Opin. Ther. Targets *20*, 869–883.

32. Fehrholz, M., Seidenspinner, S., and Kunzmann, S. (2017). Expression of surfactant protein B is dependent on cell density in H441 lung epithelial cells. PLoS ONE *12*, e0184556.

33. Srinivasan, B., Kolli, A.R., Esch, M.B., Abaci, H.E., Shuler, M.L., and Hickman, J.J. (2015). TEER measurement techniques for in vitro barrier model systems. J. Lab. Autom. *20*, 107–126.

34. Krause, G., Protze, J., and Piontek, J. (2015). Assembly and function of claudins: Structure-function relationships based on homology models and crystal structures. Semin. Cell Dev. Biol. *42*, 3–12.

35. Krause, G., Winkler, L., Mueller, S.L., Haseloff, R.F., Piontek, J., and Blasig, I.E. (2008). Structure and function of claudins. Biochim. Biophys. Acta *1778*, 631–645.

36. Kaarteenaho, R., Merikallio, H., Lehtonen, S., Harju, T., and Soini, Y. (2010). Divergent expression of claudin-1,-3,-4,-5 and-7 in developing human lung. Respir. Res. *11*, 59.

37. Kaarteenaho-Wiik, R., and Soini, Y. (2009). Claudin-1, -2, -3, -4, -5, and -7 in usual interstitial pneumonia and sarcoidosis. J. Histochem. Cytochem. *57*, 187–195.

38. Bousse, T., and Takimoto, T. (2006). Mutation at residue 523 creates a second receptor binding site on human parainfluenza virus type 1 hemagglutinin-neuraminidase protein. J. Virol. *80*, 9009–9016.

39. Markwell, M.A.K., Svennerholm, L., and Paulson, J.C. (1981). Specific gangliosides function as host cell receptors for Sendai virus. Proc. Natl Acad. Sci. USA *78*, 5406–5410.

40. Suzuki, Y., Suzuki, T., Matsunaga, M., and Matsumoto, M. (1985). Gangliosides as paramyxovirus receptor. Structural requirement of sialo-oligosaccharides in receptors for hemagglutinating virus of Japan (Sendai virus) and Newcastle disease virus. J. Biochem. *97*, 1189–1199.

41. Ran, F.A., Hsu, P.D., Wright, J., Agarwala, V., Scott, D.A., and Zhang, F. (2013). Genome engineering using the CRISPR-Cas9 system. Nat. Protoc. *8*, 2281–2308.

42. Shlyonsky, V., Goolaerts, A., Van Beneden, R., and Sariban-Sohraby, S. (2005). Differentiation of epithelial Na+ channel function. An in vitro model. J. Biol. Chem. *280*, 24181–24187.

43. Korbmacher, J.P., Michel, C., Neubauer, D., Thompson, K., Mizaikoff, B., Frick, M., Dietl, P., and Wittekindt, O.H. (2014). Amiloride-sensitive fluid resorption in NCI-H441 lung epithelia depends on an apical Cl(-) conductance. Physiol. Rep. *2*, e00201.

44. Güney, S., Schuler, A., Ott, A., Höschele, S., Zügel, S., Baloglu, E., Bärtsch, P., and Mairbäurl, H. (2007). Dexamethasone prevents transport inhibition by hypoxia in rat lung and alveolar epithelial cells by stimulating activity and expression of Na+-K+-ATPase and epithelial Na+ channels. Am. J. Physiol. Lung Cell. Mol. Physiol. *293*, L1332–L1338.

45. Blank, F., Rothen-Rutishauser, B.M., Schurch, S., and Gehr, P. (2006). An optimized in vitro model of the respiratory tract wall to study particle cell interactions. J. Aerosol Med. *19*, 392–405.

46. Hwang, T.C., and Sheppard, D.N. (1999). Molecular pharmacology of the CFTR Cl-channel. Trends Pharmacol. Sci. *20*, 448–453.

47. Neuhaus, W., Samwer, F., Kunzmann, S., Muellenbach, R.M., Wirth, M., Speer, C.P., Roewer, N., and Förster, C.Y. (2012). Lung endothelial cells strengthen, but brain endothelial cells weaken barrier properties of a human alveolar epithelium cell culture model. Differentiation *84*, 294–304.

48. Gras, D., Petit, A., Charriot, J., Knabe, L., Alagha, K., Gamez, A.S., Garulli, C., Bourdin, A., Chanez, P., Molinari, N., and Vachier, I. (2017). Epithelial ciliated beating cells essential for ex vivo ALI culture growth. BMC Pulm. Med. *17*, 80.

49. Young, S.L., Fram, E.K., and Larson, E.W. (1992). Three-dimensional reconstruction of tubular myelin. Exp. Lung Res. *18*, 497–504.

50. Williams, M.C., Hawgood, S., and Hamilton, R.L. (1991). Changes in lipid structure produced by surfactant proteins SP-A, SP-B, and SP-C. Am. J. Respir. Cell Mol. Biol. *5*, 41–50.

51. Van der Velden, J.L., Bertoncello, I., and McQualter, J.L. (2013). LysoTracker is a marker of differentiated alveolar type II cells. Respir. Res. *14*, 123.

52. Malacrida, L., Astrada, S., Briva, A., Bollati-Fogolín, M., Gratton, E., and Bagatolli, L.A. (2016). Spectral phasor analysis of LAURDAN fluorescence in live A549 lung cells to study the hydration and time evolution of intracellular lamellar body-like structures. Biochim. Biophys. Acta *1858*, 2625–2635.

53. Castellana, G., Castellana, G., Gentile, M., Castellana, R., and Resta, O. (2015). Pulmonary alveolar microlithiasis: review of the 1022 cases reported worldwide. Eur. Respir. Rev. *24*, 607–620.

54. Forbes, S.A., Bindal, N., Bamford, S., Cole, C., Kok, C.Y., Beare, D., Jia, M., Shepherd, R., Leung, K., Menzies, A., et al. (2011). COSMIC: mining complete cancer genomes in the Catalogue of Somatic Mutations in Cancer. Nucleic Acids Res. *39*, D945–D950.

55. Forbes, S.A., Tang, G., Bindal, N., Bamford, S., Dawson, E., Cole, C., Kok, C.Y., Jia, M., Ewing, R., Menzies, A., et al. (2010). COSMIC (the Catalogue of Somatic Mutations in Cancer): a resource to investigate acquired mutations in human cancer. Nucleic Acids Res. *38*, D652–D657.

56. Whitelaw, C.B.A., Radcliffe, P.A., Ritchie, W.A., Carlisle, A., Ellard, F.M., Pena, R.N., Rowe, J., Clark, A.J., King, T.J., and Mitrophanous, K.A. (2004). Efficient generation of transgenic pigs using equine infectious anaemia virus (EIAV) derived vector. FEBS Lett. *571*, 233–236.

OMTM, Volume *20*

Supplemental Information

A human surfactant B deficiency air-liquid interface cell culture model suitable for gene therapy applications

Altar M. Munis, Stephen C. Hyde, and Deborah R. Gill

Supplementary Materials

A

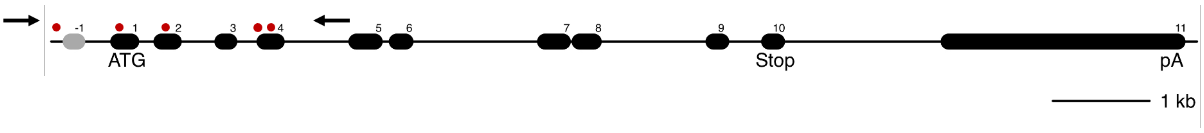

B

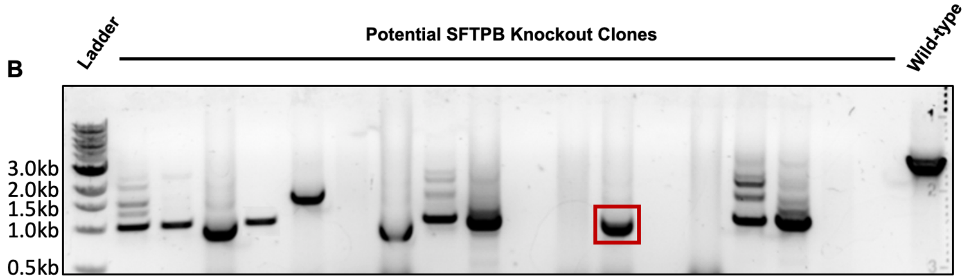

Supplementary Figure S1: Generation of SFTPB knockout H441 cells. **(A)** Schematic of human SFTPB locus. Approximate positions of exons including open reading frame initiation codon (ATG), termination codon (Stop), polyadenylation site (pA) are indicated. In several databases, an N-terminal untranslated sequence is included in the SFTPB locus (e.g. Ensembl transcript IDs ENST00000409383.6 and ENST00000393822.7). This sequence is indicated in gray as exon -1. Guide RNA target locations are indicated with red circles in numerical order (i.e. gRNA1 to gRNA5, left-to-right). The black arrows indicate the approximate locations where the primer pair used in the genotyping polymerase chain reaction bind. **(B)** A representative image of agarose gel electrophoresis following genotyping polymerase chain reaction carried out on potential *SFTPB* gene edited clones. The sizes of the DNA markers of interest are labelled. Sanger sequencing analysis revealed a large deletion encompassing the five chosen gRNAs. By removing exons -1 to 4, the coding sequence of the SPB mature homodimer was completely eliminated. The clone which was selected following sequencing is indicated with a red box. kb: kilobases.

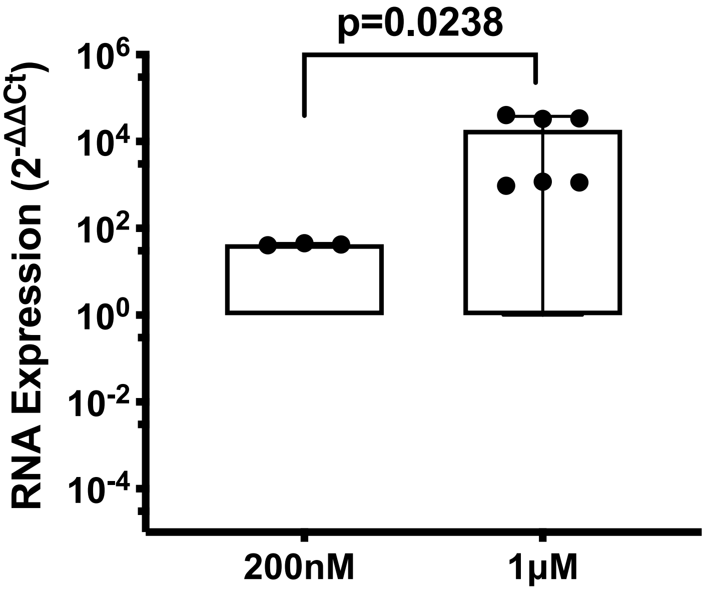

Supplementary Figure S2: Expression levels of SPB in SALI cultures supplemented with 200nM versus 1µM dexamethasone. Each data point represents a biological replicate and columns indicate mean ± SD of data shown. Mann-Whitney test was performed to compare increase in SPB mRNA levels.
